# A Novel Transposon Tn*7709* Harbors Multidrug Resistance Genes in a Pathogenic *Aeromonas media* Strain QST31

**DOI:** 10.3390/microorganisms12030572

**Published:** 2024-03-13

**Authors:** Baodi Shang, Xiaoyi Li, Xiaoping Zhang, Meiyan Zhang, Jie Kong, Jinle Wang, Aiping Tan, Feng Zhao, Defeng Zhang

**Affiliations:** 1 Guizhou Fisheries Research Institute, Guiyang 550025, China; shangbaodi1987@126.com (B.S.); lxyw2012@yeah.net (X.L.); zhangmeiyan1099@126.com (M.Z.); kellykj@126.com (J.K.); xianxue19872006@126.com (J.W.); f0328eng@126.com (F.Z.); 2 Key Laboratory of Fishery Drug Development, Ministry of Agriculture and Rural Affairs, Pearl River Fisheries Research Institute, Chinese Academy of Fishery Sciences, Guangzhou 510380, China; tanaiping@prfri.ac.cn; 3 Guangdong Provincial Key Laboratory of Aquatic Animal Immunology and Sustainable Aquaculture, Guangzhou 510380, China

**Keywords:** *Aeromonas media*, antimicrobial resistance genes, transposon, integron, tadpoles

## Abstract

Pathogenic *Aeromonas* spp. are the etiological agents of Motile Aeromonas Septicemia (MAS). This study aimed to identify the pathogen of diseased tadpoles (*Quasipaa spinosa*) and the antibiotic-resistance characteristics of this bacterium. A Gram-negative bacterium, named strain QST31, was isolated from the ascites of diseased tadpoles and was identified as *Aeromonas media* based on physiological and biochemical tests, as well as molecular identification. Artificial infection experiments showed that strain QST31 was highly virulent to tadpoles, with an LC_50_ of 2.56 × 10^7^ CFU/mL. The antimicrobial susceptibility of strain QST31 was evaluated using the disk diffusion method, and the results indicated that strain QST31 was resistant to 28 antibacterial agents. In addition, the whole genome of strain QST31 was sequenced, and the presence of antimicrobial resistance genes, integron, and transposon was investigated. Genes involved in adherence, hemolysis, type II secretion system (T2SS), T6SS, iron uptake system, and quorum sensing were identified in the genome of strain QST31. More than 12 antimicrobial resistance genes were predicted in the genome of strain QST31. Interestingly, a novel Tn*7709* transposon harboring *sul1*, *aadA16*, *catB3*, *bla*_OXA-21_, *aac*(6′)-IIa, and *tet*(A) genes was identified. In conclusion, this is the first report on the isolation and identification of pathogenic *A. media* with multidrug resistance genes from diseased tadpoles. The results revealed that preventing and controlling aquatic animal diseases caused by multidrug resistance *A. media* will be a huge challenge in the future.

## 1. Introduction

Aeromonads are known to cause severe illnesses in aquatic organisms, including fish and other cold-blooded species, and they infect humans as food-borne pathogens [1]. Motile aeromonads, including *Aeromonas hydrophila*, *A. veronii*, *A. caviae*, and *A. sobria*, are facultative pathogens that can infect fish, shrimp, reptiles, amphibians, and other aquatic species [2]. Generally, species including *A. hydrophila*, *A. veronii*, *A. salmonicida*, *A. sobria*, and *A. allosaccharophila* are isolated from aquatic environments, whereas species of *A. caviae*, *A. media*, *A. enteropelogenes*, *A. jandaei*, and *A. schubertii* are described as terrestrial and are mainly associated with food and human diseases [1]. *Aeromonas media* is a non-motile organism, and there are only a few reports of *A. media* associated with diseased aquatic animals [3,4,5,6,7,8], including koi carp (*Cyprinus carpio* koi), channel catfish (*Ietalurus punetaus*), bluntnose bream (*Myxocyprinus asiaticus*), rainbow trout (*Oncorhynchus mykiss*), shrimp (*Litopenaeus vannamei*), and Yesso scallop (*Patinopecten yessoensis*).

Bacterial diseases are a major disadvantage of aquaculture because aquatic animals are reared under crowded and stressful conditions [9]. Antimicrobials and chemical disinfectants are often used to prevent and control bacterial infections. Antibiotic-resistant bacteria, especially multiple antibiotic-resistant bacteria (MARB), continue to emerge due to the overuse of the drugs [10]. MARB are spreading around the world and have become one of the biggest public health risks in the 21st century, according to the World Health Organization. Notably, antibiotic resistance can be transferred between species and genera by spreading antibiotic-resistance genes (ARGs) using mobile genetic elements (MGEs), such as transposons, integrons, and plasmids [11]. MARB in aquaculture are a serious global concern because they cannot only infect humans via direct transmission through the food chain but also transfer acquired antimicrobial resistance to human pathogens via MGEs [12]. *A. caviae* strain LZSFT54 showed resistance to gentamicin, tobramycin, meropenem, aztreonam, cefotaxime, ceftazidime, ceftriaxone, and ciprofloxacin, and it harbored a novel *bla*_NDM-1_-bearing multidrug-resistance (MDR) transposon, containing *dfrA12*, *bla*_OXA-18_, *sul1* (two copies), and *mph* (A) [13]. *Aeromonas* spp. isolated from water samplings carried class 1 integrons (*intI*) and transposase genes of the Tn-3 family (*tnpA*) in the previous report [14]. Several investigations have been conducted on antibiotic-resistant *Aeromonas* spp. isolated from aquaculture, which is considered a reservoir of antimicrobial resistance determinants [15].

The pathogenicity of *Aeromonas* is regulated via virulence factors, including extracellular products, structural components, secretion systems, iron acquisition systems, and quorum sensing [16,17]. Types II (T2SS), III (T3SS), IV (T4SS), and VI (T6SS) are the common four secretion systems in *Aeromoas*, and T2SS is responsible for the secretion of toxins, such as aerolysin, protease, and amylases. T3SS is often found in pathogenic *Aeromonas*, such as *A. veronii* [18,19], *A. hydrophila* [17], and *A. salmonicida* [20], which are closely related to the virulence of pathogens. T6SS can inject effector proteins into the host cells, which plays an important role in bacterial competition [3]. Iron plays a critical role in the growth of most bacteria and contributes to their virulence and stress tolerance [21]. Pathogens possess siderophore-dependent or siderophore-independent mechanisms to obtain iron from their hosts and environments. Quorum sensing (QS) regulates gene expression in response to cell population density, which contributes to biofilm formation, antibiotic production, warming motility, and bacterial infection process [3]. Three autoinducer systems, AI-1 system, AI-2 system, and AI-3 system, were identified in *Aeromonas* and have affected biofilm formation, motility, and virulence [17].

The Chinese spiny frog (*Quasipaa spinosa*) is mainly distributed in China and Vietnam and is widely farmed in southern China because of its high commercial and nutritional value [22]. Since the 1980s in China, artificial breeding for *Q. spinosa* was launched because of the high market demand for frog meat [23]. Farming of *Q. spinosa* is limited by low fertilization and hatching rates, high overwinter mortality, inbreeding depression, and disease [23]. Outbreaks of various diseases, especially bacterial diseases, have hampered the *Q. spinosa* breeding industry. For example, artificial breeding of *Q. spinosa* infected with *A. hydrophila*, *Citrobacter braakii*, and *Elizabethkingia miricola* [24]. Antimicrobial drugs, including sulfonamides, enrofloxacin, amoxicillin, and doxycycline, are often used to prevent and control bacterial diseases in frog aquaculture in China. Excessive usage of antimicrobial drugs can lead to the production and spread of antibiotic-resistant bacteria. However, to date, there are no reports on the multidrug-resistant *A. meida* strain isolated from diseased *Q. spinosa*.

In this study, a Gram-negative bacterium isolated from diseased *Q. spinosa* was identified as *A. media* based on biochemical properties and molecular identification. Pathogenicity and median lethal concentration (LC_50_) were determined in healthy *Q. spinosa* using an immersion challenge. Antimicrobial susceptibility testing revealed that this strain was a multidrug-resistant bacterium. ARGs and MGEs were identified via genome sequence analysis. To the best of our knowledge, this is the first report of the isolation, identification, and molecular characterization of a multiple drug-resistant strain of *A. media* from diseased *Q. spinosa*. The results of this study provide insights into the prevention of bacterial infections in tadpoles and enhance our knowledge of the multiple drug resistance of *A. media*.

## 2. Materials and Methods

### 2.1. Clinical Signs and Bacterial Isolation

An outbreak of disease tadpoles occurred on a farm in Tongren City, Guizhou Province, China. The clinical signs of the diseased tadpoles were swollen abdomen with ascites, anal dilatation with hyperemia, and hemorrhage in the body cavity (Figure S1). The ascites of tadpoles (N = 6) were collected to streak on Tryptic Soy Agar (TSA) plates. Then, they were incubated at 28 °C for 24 h. A total of 18 isolates were purified and stored at −80 °C.

### 2.2. Identification of Isolates

The 18 isolates were streaked on TSA plates at 28 °C for 24 h, and biochemical and physiological tests were performed according to the manufacturer’s instructions for the corresponding reagents (Hangzhou Microbial Reagent Co., Ltd., Hangzhou, China).

The 16S rRNA and *gyrB* genes of these isolates were analyzed for further identification. Genomic DNA was extracted from the isolates using a Wizard Genomic DNA Purification Kit (Promega Co., Madison, WI, USA) according to the manufacturer’s instructions. PCR amplification of 16S rRNA and *gyrB* genes was performed as previously described [25] with modifications. The amplification program was as follows: pre-denaturation at 95 °C for 5 min, followed by 35 cycles of denaturation at 94 °C for 30 s, annealing at 54 °C or 55 °C for 30 s, and extension at 72 °C for 1.5 min, and final extension at 72 °C for 10 min. Amplified products were collected and sequenced by Sangon Biotech (Shanghai) Co., Ltd., Shanghai, China. The primers used in this study are listed in Table S1.

Taxonomic identification of the sequences was performed using the GenBank database (http://blast.ncbi.nlm.nih.gov/ (accessed on 20 August 2023)). The sequences of the related species were obtained from the databases. Multiple sequence alignments of the 16S rRNA and *gyrB* genes were performed using CLUSTAL W 1.81 software, and phylogenetic trees were constructed using the MEGA 5.05 software using the neighbor-joining method.

### 2.3. Challenge Test

Healthy tadpoles (35-day-old, body weight = 0.87 ± 0.02 g) with no history of disease were obtained from a frog farm in Tongren City and maintained in recirculating aquaria for 14 d to allow them to acclimatize to the environment. A total of 180 tadpoles were randomly divided into six groups, and each group was further divided into three sub-groups (N = 10 each) with three biological replicates. Tadpoles (N = 10 per tank) were cultured in 4.5 L of water in a tank (28 cm × 15 cm × 15 cm), and the water temperature was maintained at 25 °C during the experiment. The tadpoles in the experimental groups were immersion-challenged for 15 min with the representative strain QST31 at final concentrations of 1.0 × 10^9^ CFU/mL, 1.0 × 10^8^ CFU/mL, 1.0 × 10^7^ CFU/mL, 1.0 × 10^6^ CFU/mL, and 1.0 × 10^5^ CFU/mL (approximately 100 mL of bacterial suspension). The tadpoles in the control group were treated with an equal volume of phosphate-buffered saline. Clinical signs and mortality were recorded daily for 7 d post-challenge. Moribund tadpoles (N = 6) were subjected to laboratory examinations, and the ascites were selected for bacterial isolation. LC_50_ was calculated after 7 d of infection using Karber’s method.

### 2.4. Antimicrobial Susceptibility Test

The antimicrobial susceptibility test for strain QST31 was performed using the Kirby–Bauer disk diffusion assay according to the guidelines of the Clinical and Laboratory Standards Institute (CLSI, 2020) [26]. Strain QST31 was incubated in Müller Hinton Agar (MHA) at 28 °C for 18 h, and the concentration of bacterial cells was adjusted to 0.5 McFarland standard using a colorimeter. The bacterial suspension was streaked on MHA plates (Difco, Detroit, MI, USA).

The drugs included gentamicin (10 μg), streptomycin (10 μg), tobramycin (10 μg), kanamycin (30 μg), neomycin (30 μg), spectinomycin (100 μg), amikacin (30 μg), clarithromycin (15 μg), erythromycin (15 μg), doxycycline (30 μg), tetracycline (30 μg), sulfamethoxazole/trimethoprim (23.75/1.25 μg), sulfamethoxazole (300 μg), ofloxacin (5 μg), ciprofloxacin (5 μg), enrofloxacin (5 μg), norfloxacin (10 μg), florfenicol (30 μg), chloramphenicol (30 μg), cefalotin (30 μg), cefazolin (30 μg), cefotaxime (30 μg), cefoperazone (30 μg), cefixime (5 μg), ampicillin (10 μg), amoxycillin (10 μg), piperacillin (100 μg), clindamycin (2 μg), lincomycin (2 μg), vancomycin (30 μg), bacitracin (0.04 U), polymyxin B (300 IU), furantoin (300 μg), rifampicin (5 μg), and metronidazole (5 μg) antibiotic discs were selected for testing. The antibiotic disks were obtained from Hangzhou Microbial Reagent Co., Ltd. (Hangzhou, China). *Escherichia coli* strain ATCC 25,922 was used for quality control. The results were evaluated as susceptible (S), intermediate (I), or resistant (R), based on the interpretative criteria of the CLSI, 2020 [26], and manufacturer’s instructions.

### 2.5. Genome Sequencing and Function Annotation

The whole-genome sequence of strain QST31 was sequenced using a combination of Illumina HiSeq and PacBio technologies. Genome assembly was performed using the ABySS v2.0.2 (http://www.bcgsc.ca/platform/bioinfo/software/abyss, accessed on 22 November 2018), Canu v1.7 (https://github.com/marbl/canu, accessed on 22 November 2018), and GapCloser v1.12 (https://sourceforge.net/projects/soapdenovo2/files/GapCloser/, accessed on 22 November 2018). The rRNA and tRNA genes were predicted using RNAmmer-1.2 and tRNAscan-SE v1.3.1 software, respectively. The coding genes were predicted using online Rapid Annotation Subsystem Technology (RAST) (http://rast.nmpdr.org/, accessed on 26 November 2018).

#### 2.5.1. ANI, DDH, and Phylogeny Analysis

The average nucleotide identity (ANI) cutoff value of >95% and DNA–DNA hybridization (DDH) cutoff value of >70% for strains that were considered to belong to the same species. The ANI values between strain QST31 and its related Aeromonas spp. were obtained using the ANI calculator (https://www.ezbiocloud.net/tools/ani, accessed on 5 October 2023) [27]. The DDH values between the strain QST31 and the reference strains were analyzed using Genome-to-Genome Distance Calculator 3.0 (https://ggdc.dsmz.de/ggdc.php, accessed on 5 October 2023) [28].

The Genome BLAST Distance Phylogeny (GBDP) tree was constructed using FastME 2.1.6.1 and is available on TYGS (https://tygs.dsmz.de/, accessed on 5 October 2023) [28], and the figure of the GBDP tree (whole-genome sequence-based) was obtained.

#### 2.5.2. Screening of ARGs, Integron, and Novel Transposon Elements

Antimicrobial resistance genes were predicted using the Comprehensive Antibiotic Resistance Database (CARD) (https://card.mcmaster.ca/analyze, accessed on 21 October 2023). Integrons were screened in the genome of strain QST31 using Galaxy Pasteur-Integron Finder (https://galaxy.pasteur.fr/, accessed on 21 October 2023), and the sequence of integrons was obtained. The integron sequence was compared with the reference sequences in the GenBank database using online Blastn.

Transposon elements (e.g., transposase, resolvase, and relaxase) were identified using ISfinder (https://www-is.biotoul.fr/blast.php, accessed on 8 October 2023) and Blastn. The new Tn number was registered online (http://transposon.lstmed.ac.uk/, accessed on 9 October 2023).

#### 2.5.3. Virulence Factors

In the genus of *Aeromonas*, virulence factors, including hemolysins, proteases, lipases, flagella, fimbriae, lipopolysaccharides (LPS), outer membrane proteins (OMPs), secretion systems, associated toxins, iron acquisition systems, and quorum sensing communication, contribute to the strain’s pathogenicity [16]. Genes associated with virulence factors were predicted using a VFanalyzer based on the VFDB database (http://www.mgc.ac.cn/VFs/search_VFs.htm, accessed on 10 November 2023), and some of these genes were observed via sequence alignment analysis with the reference genes in the NCBI database.

## 3. Results

### 3.1. Physiological and Biochemical Characteristics

In this study, the colony morphology of the isolates from ascites was similar, appearing creamy, shiny, smooth, round, raised, and 1–2 mm in diameter after incubation on TSA plates at 28 °C for 24 h. The representative strain QST31 was non-motile, gram-negative, and positive for phenylalanine deaminase, arginine dihydrolase, oxidase, methyl-red, citrate, malonate, gelatin liquefaction, and O/129(R), and negative for H2S, ornithine decarboxylase, lysine decarboxylase, Voges-Proskauer, urea, indole test, and D-glucose. Strain QST31 could utilize D-glucose, D-mannitol, D-mannose, galactose, glycerol, arabinose, cellobiose, and maltose. The results showed that the physiological and biochemical characteristics of strain QST31 were similar to those of *A. media*.

### 3.2. Molecular Identification

**Figure 1:**
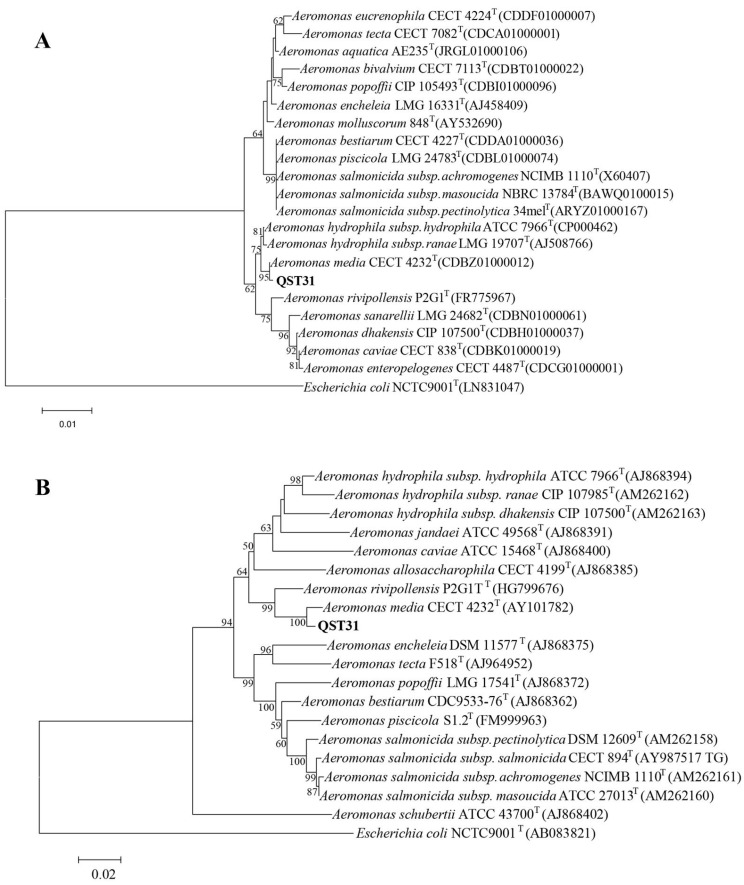
Phylogenetic trees were constructed based on the 16S rRNA (**A**) and *gyrB* (**B**) genes, showing that strain QST31 belongs to *A. media*. The GenBank accession number of the reference strains is provided in the figures. The number at each branch point is the percentage supported by bootstrap.

**Figure 2:**
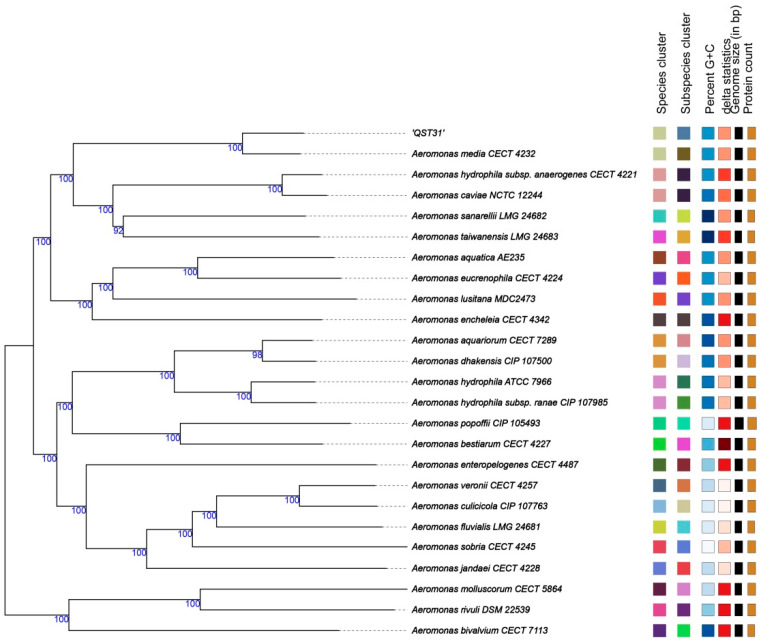
The Genome BLAST Distance Phylogeny (GBDP) tree was constructed based on the whole-genome sequence, showing that strain QST31 belongs to *A. media*. The numbers above the branches are GBDP pseudo-bootstrap support values > 60% from 100 replications, with an average branch support of 99.5%. The tree was rooted at the midpoint.

Blastn showed that the 16S rRNA sequences of strain QST31 compared with reference *A. media* strains R25-3, T5-8, and WS were 99.74%, 99.74%, and 99.81%, respectively; the *gyrB* sequences of strain QST31 compared with *A. media* strains R25-3, T5-8, and WS were 98.56%, 98.56%, and 98.45%, respectively. Phylogenetic trees of the 16S rRNA and *gyrB* genes suggested that strain QST31 and the *A. media* reference strains clustered into the same branch (Figure 1), indicating that strain QST31 belongs to *A. media*. The GBDP tree also suggested that strain QST31 clustered into *A. media* based on the whole-genome sequence (Figure 2).

Additionally, the complete genome of strain QST31 had 97.05% to 97.33% OrthoANIu with the related *A. media* species, whereas 83.92% to 93.29% OrthoANIu with other *Aeromonas* species (Table 1), which was below the cut-off threshold of 95%, indicating that strain QST31 belongs to *A. media*. In addition, DDH is an excellent method used as the taxonomic standard for species delineation in bacteria, and if the DDH value between two bacteria is below 70%, they are considered distinct species [29]. The DDH values were calculated using formula 2 of the GGDC, which indicated that strain QST31 was closely related to *A. media* according to the traditional 70% DDH criterion (Table 1). In conclusion, strain QST31 was identified as *A. media* based on analysis of the 16S rRNA, *gyrB*, GBDP tree, ANI, and DDH.

### 3.3. Pathogenesis Assay

**Figure 3:**
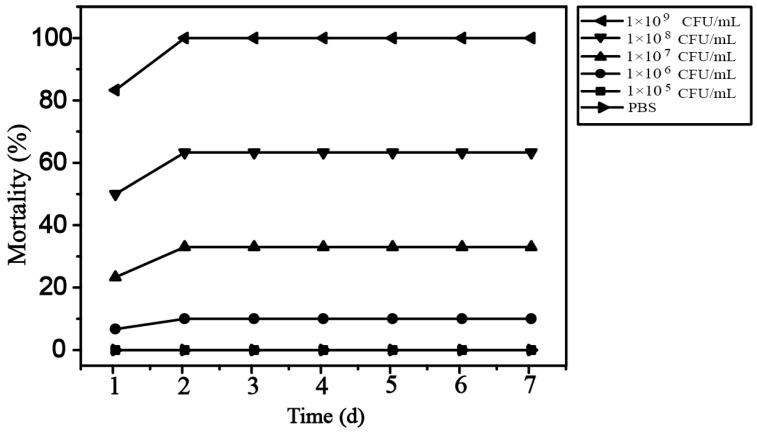
Cumulative mortality rates of tadpoles challenged with strain QST31.

The challenge test showed that healthy tadpoles infected with strain QST31 at concentrations of ≥1.0 × 10^6^ CFU/mL began to die on the first day. The cumulative mortality rates were 100.0%, 63.3%, 33.3%, 10.0%, and 0.0% for strain QST31 at the concentrations of 1.0 × 10^9^ CFU/mL, 1.0 × 10^8^ CFU/mL, 1.0 × 10^7^ CFU/mL, 1.0 × 10^6^ CFU/mL, and 1.0 × 10^5^ CFU/mL, respectively (Figure 3). The LC_50_ value was 2.69 × 10^7^ CFU/mL. The clinical signs in tadpoles infected with strain QST31 were similar to those observed in natural infections, including swollen abdomen with ascites, anal dilatation with hyperemia, and petechial hemorrhage in the body cavity. The colony morphology of the re-isolates from the ascites of infected tadpoles in the experimental groups was similar to that of strain QST31, and the similarity of 16S rRNA gene sequences between re-isolates and strain QST31 was 100%. In the control group, no clinical signs or mortalities were observed, and no bacteria were isolated from the liver and kidney.

### 3.4. Antimicrobial Susceptibility

Antimicrobial susceptibility tests showed that strain QST31 was resistant to aminoglycosides (spectinomycin, streptomycin, gentamicin, tobramycin, kanamycin, and neomycin), quinolones (ciprofloxacin and norfloxacin), tetracyclines (tetracycline and doxycycline), polypeptides (bacitracin), macrolides (clarithromycin and erythromycin), sulfonamides (sulfamethoxazole/trimethoprim and sulfamethoxazole), beta-lactams (cefalotin, cefalexin, ampicillin, amoxicillin, cefixime, cefazolin, and piperacillin), glycopeptides (vancomycin), lincosamides (clindamycin and lincomycin), and other antibiotics including rifampicin and metronidazole (Table 2 and Figure S2), indicating that it is a multidrug-resistant strain. However, strain QST31 was sensitive to several antimicrobial drugs, including amikacin, ofloxacin, and furantoin. In terms of quinolones, strain QST31 showed a slight difference in the diameter of the inhibition zone for ofloxacin, ciprofloxacin, and enrofloxacin; however, there is a significant difference in the phenotypes, which may be related to different breakpoints values or resistance mechanism.

### 3.5. Genome and Function Annotation

The complete genome sequence of strain QST31 was 4,605,106 bp, with a GC content of 61.13% (GenBank accession no. CP137009.1). Genome annotation showed that a total of 4023 coding genes and 158 RNAs (127 tRNAs, 10 23S rRNAs, 10 16S rRNAs, and 11 5S rRNAs) were predicted.

#### 3.5.1. ARGs and Class 1 Integrons

**Figure 4:**
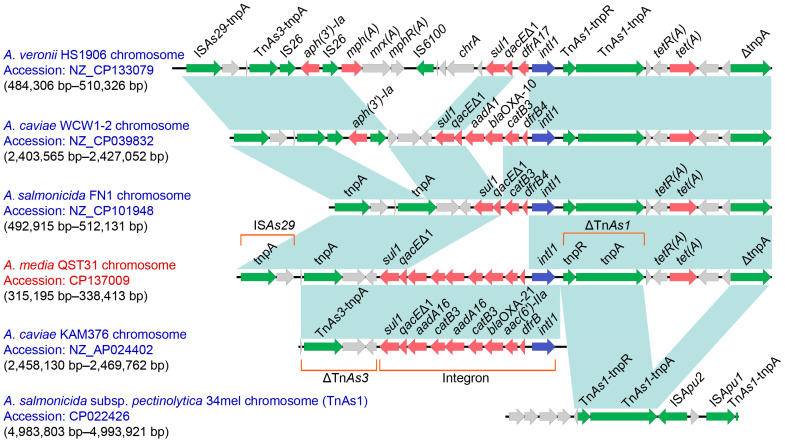
Genetic features of a novel Tn*7709*. The physical maps were generated using Easyfig 2.2.3. Genetic structures of Tn*7709* in the genome of *A. media* QST31 and comparison to related regions from *A. veronii* HS1906 (CP133079), *A. caviae* WCW1-2 (CP039832), *A. salmonicida* FN1 (CP101948), *A. caviae* KAM376 (AP024402), and *A. salmonicida* subsp. *pectinolytica* 34mel (CP022426). Genes are displayed by arrows. Resistance genes and mobile genetic elements are highlighted in red and green, respectively. Regions of >99% identity are shown by blue shading. Δ truncates genes or regions. ARO, Antibiotic Resistance Ontology.

A total of 14 antimicrobial resistance genes (including two copies of the *catB3* and *aadA16* genes) were predicted using the CARD database with strict hit criteria, including *bla*_OXA-917_, *sul1*, *aadA16*, *catB3*, *bla_OXA_*_-21_, aac(6′)-IIa, *tet*(A), *bla*_MOX-9_, *adeF*, *vanT*, and EF-Tu mutants (Table 3). Furthermore, the coding genes of *IntI*1, *aac*(6′)-IIa, *bla*_OXA-21_, *catB3*, *aadA16*, *catB3*, *aadA16*, *qacE*Δ1, and *sul1* located in the same gene cassette were identified (Figure 4), with a 5′ conserved segment (5′ CS) (*intI1*) and a 3′ CS (*qacE*Δ1-*sul1*). This gene cassette was identified as class 1 integron and displayed 100% query coverage and 99% identity to reference strain *A. caviae* KAM376, corresponding to bases 2,458,130 to 2,469,762 in GenBank accession no. AP024402.1.

Additionally, in the quinolone resistance determining regions (QRDR) of GyrA, the substitution of Ser-83-Ile was detected in strain QST31, and the substitution of Ser-80-Ile was found in the QRDR of ParC, and these substitutions conferred resistance to quinolone antibiotics. In addition, the *macB* gene associated with macrolide antibiotic resistance was predicted using loose hit criteria, the RpoB mutant (S531G, H526S, D516G, L511R, and N518I) associated with rifamycin resistance, *bacA* associated with peptic resistance, and *mcr-7.1* related to colistin resistance were predicted in the genome, and the *tetR*, *msbA*, *dfrA3*, *vatF*, and *fusA* genes were also predicted using loose hit criteria with best identifies more than 50 (Table S2).

#### 3.5.2. Novel Transposon

Sequence analysis indicated that 10 resistance genes, including *sul1*, *qacE*Δ1, *aadA16*, *catB3*, *aadA16*, *catB3*, *bla*_OXA-21_, *aac*(6′)-Ⅱa, *dfrB*, and *tet*(A) genes, were carried by a novel IS*As29* composite transposon designated Tn*7709* (Figure 4), according to the nomenclature of transposons. Tn*7709* is 23,218 bp in length, corresponding to bases 315,195 to 338,413 in the genome of QST31 (GenBank accession no. CP137009.1). The structures of Tn*7709* were surrounded by IS*As29*-*tnpA* (located upstream) and truncated Δ*tnpA* (located downstream). Compared to *A. media* QST31 and *A. salmonicida* FN1 strains, *A. veronii* HS1906 and *A. caviae* WCW1-2 strains carried *aph*(3′)-Ⅰa and IS*26* in the downstream of Tn*As3*. Although *A. veronii* HS1906, *A. caviae* WCW1-2, and *A. salmonicida* FN1 also carried class 1 integrons, their gene cassettes were different from those of strains QST31 and KAM376. Tn7709 is likely derived from the insertion of Tn*As3* transposase and class 1 integron into the complex transposon between IS*As29* and Tn*As1* carrying *tet*(A).

#### 3.5.3. Virulence Factors

The virulence factors of strain QST31 were analyzed using the VFDB database and online Blastn/Blastp (Table S3).

Adherence: Pilus and flagellar associated with adherence and motility were predicted in strain QST31, including Flp type Ⅳ pili, Mannose-sensitive hemagglutinin (Msh) plus, Polar flagella, Tap type Ⅳ pili, and LPS O-antigen. In addition, encoding genes of OMPs, including *ompH*, *ompK*, and *ompW*, were identified.

Secretion system: The secretion systems T2SS and T6SS were found in the genome of strain QST31, whereas T3SS was absent in strain QST31.

Toxin: Three toxin factors were detected in strain QST31, including hemolysin HlyA, thermostable hemolysin (TSH), and thermostable hemolysin Ⅲ. Common toxin factors such as aerolysin (AerA), cytotoxic enterotoxin (Act), heat-stable cytotoxic enterotoxin (Ast), extracellular hemolysin (Ahh1), and the repeat in toxin (RTX) were absent in this strain.

Iron acquisition: The iron uptake-associated genes such as *fstC* (encoding FstC), the biosynthetic gene cluster of amonabactin [30], and encoding genes of heme uptake and heme receptor proteins were found in strain QST31.

Quorum sensing: The encoding genes of LuxS, AhyR, AhyI, QseB, and QseC associated with quorum sensing systems (AI-1, AI-2, and AI-3) were identified in the genome of strain QST31, according to previous reports [17,31]. 

## 4. Discussion

The genus *Aeromonas* comprises 36 species and is widely distributed in various aquatic environments. Important fish pathogens, such as *A. hydrophila*, *A. salmonicida*, *A. veronii*, *A. sobria*, *A. schubertii*, *A. piscicola*, and *A. dhakensis* are frequently isolated from fish, whereas *A. allosaccharophila*, *A. dhakensis*, *A. caviae*, *A. veronii*, *A. hydrophila*, *A. jandaei*, *A. media*, and *A. trota* are often isolated from eels [3]. Identification of *Aeromonas* at the species level using phenotypic characterization alone is difficult, and molecular identification is necessary for accurate distinction of *Aeromonas* at the species level [1,2,3,32]. Genes such as 16S rRNA, *gyrB*, *rpoD*, and genome sequence analysis based on ANI and DDH are commonly used for species delineation. Strain QST31 was identified as *A. media* based on physiological and biochemical characteristics, sequence analysis of the 16S rRNA and *gyrB* genes, and analysis of ANI and DDH at the genome level.

*A. media* was first isolated from river freshwater and is often isolated from sewage water, activated sludge, drinking water, earthworm gut content, chicken, animals, and humans [32,33]. It is an opportunistic pathogen that causes serious diseases in aquatic organisms (fish and other cold-blooded species) and humans, particularly as foodborne pathogens [4,32,34]. Strain QST31 was isolated from diseased tadpoles and had an LC_50_ of 2.69 × 10^7^ CFU/mL from bathing infection, indicating that this strain was highly virulent to tadpoles. *A. media* is an emerging pathogen for tadpoles, which poses a public health implication as a foodborne pathogen.

In recent decades, the genus *Aeromonas* has received increasing attention owing to its opportunistic pathogenicity and resistance to multiple antibiotics [15,35]. Antimicrobial drugs have long been used to control bacterial diseases in aquaculture. The overuse of antimicrobial drugs has led to the emergence of antimicrobial-resistant and multidrug-resistant strains worldwide [36]. *Aeromonas* from aquaculture has shown an increasing tendency to develop resistance to various classes of antibiotics, posing risks to water quality and human health. In recent years, an increase in the antimicrobial resistance of *Aeromonas* spp. strains have been observed in fish farms [37,38]. A multi-drug resistance *A. media* strain QST31 was isolated from tadpoles and showed significant resistance to aminoglycosides, quinolones, tetracyclines, polypeptides, macrolides, sulfonamides, beta-lactams, glycopeptides, and lincosamides. In previous reports, *A. media* strain KC-2 isolated from koi carp showed resistance to several antimicrobial drugs, such as cefalotin, cefixime, cefotaxime, gentamicin, netilmicin, azithromycin, and chloramphenicol [8]; *A. media* strain SD/21–15 isolated from marine sediments showed multidrug resistance to ampicillin, cefoxitin, cephalothin, penicillin, tetracycline, and amoxicillin [4]. Strain QST31 showed resistance to more antimicrobials than other *A. media* strains in previous reports, posing a serious threat to aquatic animals and humans.

Antimicrobial resistance genes were analyzed via genome sequencing. Strain QST31 possessed at least 14 resistance genes associated with antibiotic resistance belonging to various drug classes. In this study, three *β*-lactam resistance genes (*bla*_OXA-917_, *bla*_OXA-21_, and *bla*_MOX-9_), two aminoglycosides resistance genes (*aac*(6′)-IIa and *aadA16*), two tetracyclines resistance genes (*tetA* and *tetR*), two quinolones resistance genes (mutations on *gyrA* and *parC*), amphenicols resistance gene (*catB3*), sulfonamides resistance gene (*sul1*), trimethoprim resistance gene (*dfrA3*), glycopeptides resistance gene (*vanT*), macrolides resistance gene (*macB*), rifamycin resistance genes (*rpoB* mutant), peptides resistance gene (*bacA*), colistin resistance gene (*mcr-7.1*), and other resistance genes (*adeF*, *Ef-Tu*, and others) were identified in the genome of strain QST31. Detection of resistance genes revealed that strain QST31 carried numerous ARGs, most of which mediated resistance to the corresponding antimicrobials. The presence of *tet*(34), *mcr-7.1*, *mcr-3*, and *dfrA3* in all *A. veronii* genomes (53 strains), and *sul1* and *sul2* were detected in a few strains [39]. The *adeF* and *Ef-Tu* are the most abundant ARGs in *Aeromonas species*, and approximately 25% of *Aeromonas* strains carried *sul1*, *tet*(A), or *tet*(D) [40]. The frequencies of *bla*_OXA.917_ and *vanT* genes in *A. media* strains of aquatic animals were higher than those of terrestrial animals, with 58% and 95%, respectively [40]. Strain QST31 carries a large number of ARGs, which are prevalent in *Aeromonas*, and these ARGs can be disseminated to other bacteria through horizontal gene transfer. Therefore, *A. media* QST31 is an important consideration as a potential reservoir of ARGs in aquatic animals.

Integrons are natural gene capture systems that play an important role in the dissemination of resistance genes. Class 1 integron contains an integrase gene (*intI 1*) that corresponds to the 5′ CS, a variable region size where cassettes are located, a sulfonamide resistance gene (*sul1*) that corresponds to the 3′ CS, and a quaternary ammonium compound resistance sequence (*qacE*Δ1) [41]. Class 1 integrons are the most common integron type and are found in various Gram-negative bacteria. Class 1 integrons in the *Aeromonas* species often carry various antimicrobial resistance gene cassettes [36,42]. However, limited information is available on the class 1 integrons that contain resistance genes in *A. media*. Class 1 integron was identified in strain QST31 using Blastn in the GenBank database. The class 1 integron in strain QST31 harbored the *aacA4* gene cassette, *bla_OXA-2_* gene cassette, *catB3* gene cassette, *aadA16* gene cassette, and a combination of the *aadA16* and *catB3* gene cassettes, which correspond to resistance to aminoglycosides, *β*-lactam antibiotics, and chloramphenicol. A novel Tn*7709* was identified through whole genome sequence analysis, and this transposon contained 10 resistance genes, including *sul1*, *qacE*Δ1, *aadA16*, *catB3*, *aadA16*, *catB3*, *bla*_OXA-21_, *aac*(6′)-IIa, *dfrB*, and *tet*(A) genes. These results suggest that the resistance genes carried by Tn*7709* have the potential for horizontal transfer to other bacteria. The presence of a novel transposon indicates that strain QST31 poses a public health problem.

Secretion systems for T2SS, T3SS, T4SS, and T6SS are frequently present in *Aeromonas* species [16]. In previous studies, all *A. media* strains possessed a T2SS, whereas only a few strains had *vrG*1, *vgrG*3, *hcp*, and *ats* genes with an incomplete T6SS [4]. The T6SS is a Sec-independent secretory system that transports proteins directly to the cell surface or host cells [3]. The T2SS is an exclusive secretory system essential for the pathogenesis of *A. hydrophila* and *A. salmonicida*. Genes encoding the T2SS and T6SS were detected in the genome of strain QST31, indicating that this strain could secrete effectors or virulent factors to damage host cells and have stronger adaptability to the environment and host. Furthermore, numerous virulence factors involved in adherence (Msh pilus, polar flagella, Tap type Ⅳ pili, and LPS O-antigen), toxins (hemolysin HlyA, hemolysin III, and thermostable hemolysin TSH), immune evasion (capsule), serum resistance (*rmlD*), and stress adaptation (*katG*) have been identified in the genome. Consistent with a previous report, strain QST31 and 25 other *A. media* strains harbored three hemolysin genes (*hlyA*, *hlyⅢ*, and *TSH*), which form cytotoxic enterotoxins, causing membrane damage and fluid accumulation in host cells, whereas none were found the *aerA* gene [3]. In addition, genes associated with AI-1, AI-2, and AI-3 were found, indicating that strain QST31 had comprehensive QS systems, which could regulate virulence, motility, and biofilm. Genes related to iron uptake systems were identified, which contribute to the virulence of strain QST31 in tadpoles. The genome sequence of strain QST31 provides insight into the pathogenesis of *A. media*.

## 5. Conclusions

In conclusion, strain QST31 was isolated from diseased tadpoles and identified as *A. media* based on physiological and biochemical characteristics and molecular identification. Strain QST31 exhibited multidrug resistance and harbored ≥ 12 ARG genes, with a novel Tn*7709* identified based on genome sequence analysis. Furthermore, strain QST31 is highly virulent to tadpoles and carries many virulence-related genes, including coding genes associated with hemolysins, adherence, catalase-peroxidase, toxins, secretion systems, iron uptake, and quorum sensing. To our knowledge, this is the first report of *A. media* carrying an intact T6SS and a novel Tn*7709* transposon. This study provides new insights into the pathogenesis and horizontal transfer of ARG genes in *A. media*. Thus, environmentally friendly control strategies should be encouraged to reduce the spread of multidrug-resistant strains.

## Figures and Tables

**Table 1:** Comparison of ANI and DDH values between the genome sequence of strain QST31 and those of related species.

Species (GeneBank Accession No.)	OrthoANIu Value (%)	DDH Value (%)
*Aeromonas media* strain E31 (CP067417)	97.06	74.6
*Aeromonas media* strain K521 (CP118993)	97.05	74.4
*Aeromonas media* strain TR3_1 (CP075564)	97.33	77.5
*Aeromonas rivipollensis* strain KN-Mc-11N1 (CP027856)	93.29	52.1
*Aeromonas caviae* KAM497 (AP026375)	88.20	35.1
*Aeromonas caviae* strain CYEY0630 (CP118442)	88.15	34.9
*Aeromonas encheleia* strain H4-C21 (CP093843)	87.55	33.6
*Aeromonas hydrophila* strain AH10 (CP011100)	86.61	31.8
*Aeromonas hydrophila* strain AC185 (CP093308)	86.55	31.8
*Aeromonas salmonicida* strain AS3 (CP110645)	85.13	29.7
*Aeromonas veronii* strain X11 (CP024930)	84.32	28.7
*Aeromonas veronii* strain MS-18-37 (CP033604)	84.28	28.7
*Aeromonas jandaei* strain GTBM29 (CP046270)	83.92	27.9
*Aeromonas jandaei* strain GTBM29 (CP046270)	83.92	27.9

Notes: ANI, average nucleotide identity; DDH, DNA–DNA hybridization.

**Table 2:** Antimicrobial susceptibility patterns of strain QST31.

Drug Classification	No.	Antimicrobial Drugs	Breakpoints (mm)			Inhibition Zone (mm)	Sensitivity
			R	I	S		
Aminoglycosides	1	Gentamicin	≤12	13~14	≥15	11	R
	2	Streptomycin	≤11	12~14	≥15	6	R
	3	Tobramycin	≤12	13~14	≥15	6	R
	4	Kanamycin	≤13	14~17	≥18	6	R
	5	Neomycin	≤12	13~16	≥17	11	R
	6	Spectinomycin	≤9	10~15	≥16	6	R
	7	Amikacin	≤14	15~16	≥17	18	S
Macrolides	8	Clarithromycin	≤13	14~17	≥18	11	R
	9	Erythromycin	≤13	14~22	≥23	12	R
Tetracyclines	10	Doxycycline	≤12	13~15	≥16	9	R
	11	Tetracycline	≤14	15~18	≥19	6	R
Sulfonamides	12	Sulfamethoxazole/ trimethoprim	≤9	10~15	≥16	6	R
	13	Sulfamethoxazole	≤9	10~15	≥16	6	R
Quinolones	14	Ofloxacin	≤12	13~15	≥16	16	S
	15	Ciprofloxacin	≤15	16~20	≥21	15	R
	16	Enrofloxacin	≤15	16~20	≥21	16	I
	17	Norfloxacin	≤12	13~16	≥17	9	R
Amphenicols	18	Florfenicol	≤12	13~17	≥18	14	I
	19	Chloramphenicol	≤12	13~17	≥18	9	R
*β*-lactam	20	Cefalotin	≤14	15~17	≥18	7	R
	21	Cefazolin	≤14	15~17	≥18	6	R
	22	Cefotaxime	≤14	15~22	≥23	14	R
	23	Cefoperazone	≤15	16~20	≥21	16	I
	24	Cefixime	≤9	10~15	≥16	8	R
	25	Ampicillin	≤9	10~15	≥16	6	R
	26	Amoxicillin	≤13	14~17	≥18	6	R
	27	Piperacillin	≤17	18~20	≥21	6	R
Lincosamides	28	Clindamycin	≤14	15~20	≥21	7	R
	29	Lincomycin	≤9	10~15	≥16	6	R
Glycopeptides	30	Vancomycin	≤9	10~15	≥16	6	R
Polypeptides	31	Bacitracin	≤8	9~12	≥13	6	R
Others	32	Polymyxin B	≤8	8~11	≥12	11	I
	33	Furantoin	≤14	15~16	≥17	17	S
	34	Rifampicin	≤16	17~19	≥20	10	R
	35	Metronidazole	≤9	10~15	≥16	6	R

Notes: R, Resistant; I, Intermediate; S, Susceptible.

**Table 3:** Predicted antimicrobial resistance genes (cut off with strict and perfect) in the genome of the QST31 strain.

ARO Name	ARO Accession	Position	Orientation	AMR Gene Family	Drug Class	Resistance Mechanism
*bla*_OXA-917_	3006103	31,390–32,184	−	OXA beta-lactamase	carbapenem; cephalosporin; penam	antibiotic inactivation
*sul1*	3000410	321,390–322,229	−	sulfonamide resistant sul	sulfonamide antibiotic	antibiotic target replacement
*aadA16*	3002616	322,586–323,431	-	ANT(3″)	aminoglycoside antibiotic	antibiotic inactivation
*catB3*	3002676	323,590–324,222	−	chloramphenicol acetyltransferase (CAT)	phenicol antibiotic	antibiotic inactivation
*aadA16*	3002616	324,206–325,069	−	ANT(3″)	aminoglycoside antibiotic	antibiotic inactivation
*catB3*	3002676	325,228–325,860	−	chloramphenicol acetyltransferase (CAT)	phenicol antibiotic	antibiotic inactivation
*bla*_OXA-21_	3001416	325,922–326,749	−	OXA beta-lactamase	carbapenem; cephalosporin; penam	antibiotic inactivation
*aac*(6′)-IIa	3002594	326,819–327,373	−	AAC(6′)	aminoglycoside antibiotic	antibiotic inactivation
*tet*(A)	3000165	333,905–335,179	+	major facilitator superfamily (MFS) antibiotic efflux pump	tetracycline antibiotic	antibiotic efflux
*bla*_MOX-9_	3002191	1,301,154–1,302,305	+	MOX beta-lactamase	cephalosporin; cephamycin; penam	antibiotic inactivation
*adeF*	3000777	1,555,231–1,558,380	+	resistance-nodulation-cell division (RND) antibiotic efflux pump	fluoroquinolone antibiotic; tetracycline antibiotic	antibiotic efflux
*vanT*	3002972	3,588,886–3,589,959	−	glycopeptide resistance gene cluster; vanT	glycopeptide antibiotic	antibiotic target alteration
EF-Tu mutants	3003369	4,320,945–4,322,129	−	elfamycin resistant EF-Tu	elfamycin antibiotic	antibiotic target alteration
EF-Tu mutants	3003369	4,340,145–4,341,329	−	elfamycin resistant EF-Tu	elfamycin antibiotic	antibiotic target alteration

## Data Availability

The data presented in this study are available on request from the corresponding author.

## Supplementary Materials

The following supporting information can be downloaded at https://www.mdpi.com/article/10.3390/microorganisms12030572/s1: Table S1: Primers of the 16S rRNA and *gyrB* genes for PCR amplification; Table S2: Predicted antimicrobial resistance genes (cut off with loose and best identifies > 50) in the genome of the QST31 strain; Table S3: Virulence gene profiles of *Aeromonas media* QST31 strains (light green) and 5 reference strains (dark blue). Figure S1: Clinical signs of the naturally diseased tadpoles; Figure S2: Multidrug resistance phenotype of strain QST31 based on the Kirby–Bauer disk diffusion assay.
