# Supplementary material for: A Novel Transposon Tn7709 Harbors Multidrug Resistance Genes in a Pathogenic Aeromonas media Strain QST31
Source: Microorganisms. 2024 Mar 13;12(3):572. doi: 10.3390/microorganisms12030572 (PMC10975346; doi:10.3390/microorganisms12030572)
Supplement: Supplementary file 1 [file microorganisms-12-00572-s001.zip › microorganisms-2878904-supplementary.pdf]

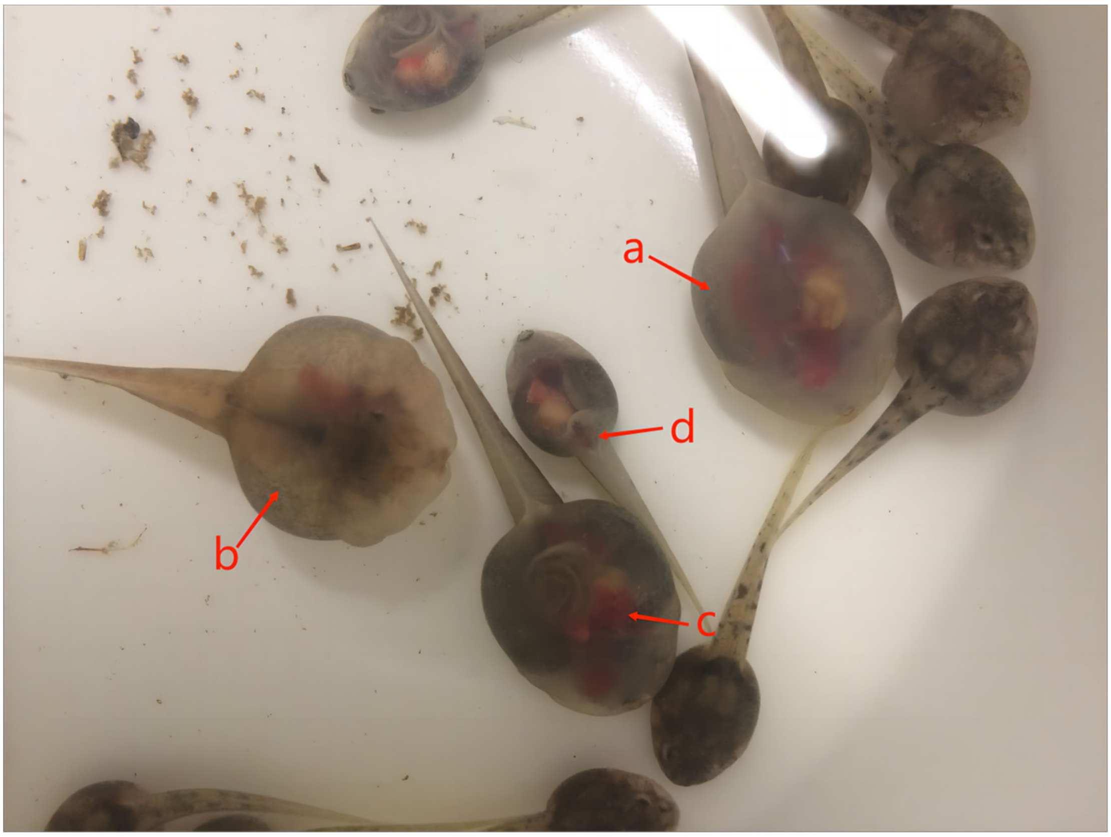

Figure S1. Clinical signs of the naturally diseased tadpoles. a and b, swollen abdomen with ascites; c, hemorrhage in the body cavity; d, anal dilatation with hyperemia.

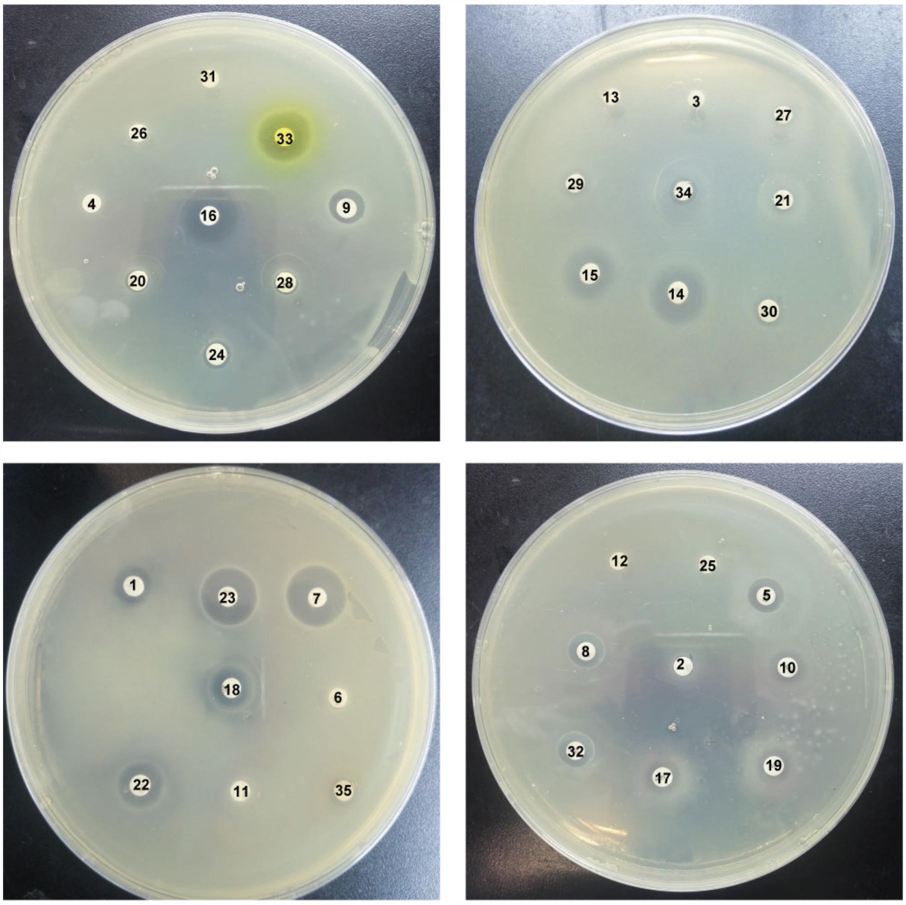

Figure S2.

Multidrug resistance phenotype of strain QST31 based on the Kirby-Bauer disk diffusion assay. The numbers in this figure are corresponding to the numbers in Table 2.

Table S1

Primers of the 16S rRNA and *gyrB* genes for PCR amplification

Genes	Primer Name	Primer Sequence (5'-3')	Annealing Temperature (°C)	Product Size (bp)
16s rRNA	27f	AGAGTTTGATCATGGCTCAG	54	1500
	1492r	GGTTACCTTGTTACGACTT		
gyrB	gyrB-3F	TCCGGCGGTCTGCACGGCGT	55	1200
	gyrB-14R	TTGTCCGGGTTGTACTCGTC		

Table S2.

Predicted antimicrobial resistance genes (cut off with loose and best identifies >50) in the genome of the QST31 strain

ARO Aame	ARO Accession	Position	Orientation	AMR Gene Family	Drug Class	Resistance Mechanism
rsmA	3005069	510,948-511,136	+	resistance-nodulation-cell division (RND) antibiotic efflux pump	fluoroquinolone antibiotic; diaminopyrimidine antibiotic; phenicol antibiotic	antibiotic efflux
CRP	3000518	3,608,993-3,609,631	+	resistance-nodulation-cell division (RND) antibiotic efflux pump	macrolide antibiotic; fluoroquinolone antibiotic; penam	antibiotic efflux
UhpT	3003890	1,440,943-1,442,349	-	antibiotic-resistant UhpT	phosphonic acid antibiotic	antibiotic target alteration
gyrA	3003294	2,385,405-2,388,194	-	fluoroquinolone resistant gyrA	fluoroquinolone antibiotic	antibiotic target alteration
bacA	3002986	4,415,200-4,416,015	+	undecaprenyl pyrophosphate related proteins	peptide antibiotic	antibiotic target alteration
mcr-7.1	3004517	3,142,787-3,144,412	-	MCR phosphoethanolamine transferase	peptide antibiotic	antibiotic target alteration
parC	3003308	466,816-469,107	+	fluoroquinolone resistant parC	fluoroquinolone antibiotic	antibiotic target alteration
yojI	3003952	11,873-13,519	-	ATP-binding cassette (ABC) antibiotic efflux	peptide antibiotic	antibiotic efflux

				pump		
katG	3003392	2,017,477-2,019,651	-	isoniazid resistant katG	isoniazid-like antibiotic	antibiotic target alteration
rosA	3003048	4,548,728-4,549,948	+	major facilitator superfamily (MFS) antibiotic efflux pump	peptide antibiotic	antibiotic efflux
msbA	3003950	1,702,361-1,704,130	+	ATP-binding cassette (ABC) antibiotic efflux pump	nitroimidazole antibiotic	antibiotic efflux
dfrA3	3003105	1,057,200-1,057,694	+	trimethoprim resistant dihydrofolate reductase dfr	diaminopyrimidine antibiotic	antibiotic target replacement
fusA	3003735	4,322,184-4,324,289	-	antibiotic resistant fusA	fusidane antibiotic	antibiotic target alteration
gyrB	3004562	4,600,326-4,602,737	-	fluoroquinolone resistant gyrB	fluoroquinolone antibiotic	antibiotic target alteration
mdtH	3001216	906,007-907,200	-	major facilitator superfamily (MFS) antibiotic efflux pump	fluoroquinolone antibiotic	antibiotic efflux
oprM	3000379	1,558,373-1,559,791	+	resistance-nodulation-cell division (RND) antibiotic efflux pump	macrolide antibiotic; fluoroquinolone antibiotic; monobactam; aminoglycoside antibiotic; carbapenem; cephalosporin; cephamycin; penam; tetracycline antibiotic; peptide antibiotic; aminocoumarin	antibiotic efflux

					antibiotic; diaminopyrimidine antibiotic; sulfonamide antibiotic; phenicol antibiotic; penem; disinfecting agents and antiseptics	
rpoB	3003283	4,332,331-4,336,359	-	rifamycin-resistant beta-subunit of RNA polymerase (rpoB)	rifamycin antibiotic	antibiotic target alteration; antibiotic target replacement
acrA	3004042	1,554,029-1,555,213	+	resistance-nodulation-cell division (RND) antibiotic efflux pump	fluoroquinolone antibiotic; cephalosporin; glycylcycline; penam; tetracycline antibiotic; rifamycin antibiotic; phenicol antibiotic; disinfecting agents and antiseptics	antibiotic efflux
H-NS	3000676	2,011,503-2,011,907	-	major facilitator superfamily (MFS) antibiotic efflux pump; resistance-nodulation-cell division (RND) antibiotic efflux pump	macrolide antibiotic; fluoroquinolone antibiotic; cephalosporin; cephamycin; penam; tetracycline antibiotic	antibiotic efflux
H-NS	3000676	1,463,249-1,463,656	+	major facilitator superfamily (MFS)	macrolide antibiotic; fluoroquinolone	antibiotic efflux

				antibiotic efflux pump; resistance-nodulation-cell division (RND) antibiotic efflux pump	antibiotic; cephalosporin; cephamycin; penam; tetracycline antibiotic	
rpoB	3007051	4,327,936-4,332,240	-	rifamycin-resistant beta-subunit of RNA polymerase (rpoB)	fluoroquinolone antibiotic; rifamycin antibiotic	antibiotic target alteration; antibiotic target replacement
ompA	3005044	4,013,749-4,014,780	+	General Bacterial Porin with reduced permeability to peptide antibiotics	peptide antibiotic	reduced permeability to antibiotic
imiS	3003095	795,461-795,619	+	CphA beta-lactamase	carbapenem	antibiotic inactivation
tetR	3003479	333,224-333,901	-	major facilitator superfamily (MFS) antibiotic efflux pump	tetracycline antibiotic	antibiotic target alteration; antibiotic efflux
vatF	3003744	1,085,937-1,086,482	-	streptogramin vat acetyltransferase	streptogramin antibiotic; streptogramin A antibiotic	antibiotic inactivation
murA	3003776	4,157,696-4,158,952	+	antibiotic-resistant murA transferase	phosphonic acid antibiotic	antibiotic target alteration
macB	3000535	804,149-806,098	-	ATP-binding cassette (ABC) antibiotic efflux pump	macrolide antibiotic	antibiotic efflux
cpxR	3004054	162,336-163,028	-	resistance-nodulation-cell division (RND) antibiotic efflux pump	macrolide antibiotic; fluoroquinolone antibiotic; monobactam; aminoglycoside	antibiotic efflux

					antibiotic; carbapenem; cephalosporin; cephamycin; penam; tetracycline antibiotic; peptide antibiotic; aminocoumarin antibiotic; diaminopyrimidine antibiotic; sulfonamide antibiotic; phenicol antibiotic; penem	
golS	3000504	4,504,321-4,504,710	-	resistance-nodulation-cell division (RND) antibiotic efflux pump	monobactam; carbapenem; cephalosporin; cephamycin; penam; phenicol antibiotic; penem	antibiotic efflux
tolC	3000237	459,350-460,651	-	ATP-binding cassette (ABC) antibiotic efflux pump; major facilitator superfamily (MFS) antibiotic efflux pump; resistance-nodulation-cell division (RND) antibiotic efflux pump	macrolide antibiotic; fluoroquinolone antibiotic; aminoglycoside antibiotic; carbapenem; cephalosporin; glycylcycline; cephamycin; penam; tetracycline antibiotic;	antibiotic efflux

					peptide antibiotic; aminocoumarin antibiotic; rifamycin antibiotic; phenicol antibiotic; penem; disinfecting agents and antiseptics	
tet(35)	3000481	2,012,485-2,014,065	+	ATP-binding cassette (ABC) antibiotic efflux pump	tetracycline antibiotic	antibiotic efflux

Table S3

Virulence gene profiles of *Aeromonas media* QST31 strains (light green) and 5 reference strains (dark blue)

VFclass	Virulence Factors	Related Genes	*A. media* QST31	A. hydrophila ML09-119	A. hydrophila subsp. hydrophila ATCC 7966	A. salmonicida subsp. salmonicida A449		A. veronii B565
			QST31 (QST31)	Chromosome (NC_021290)	Chromosome (NC_008570)	Chromosome (NC_009348)	5 (NC_009350)	Chromosome (NC_015424)
Adherence	Flp type IV pili	flp1	-	AHML_08075	AHA_1450	ASA_2915	-	B565_2735
		flpA	-	AHML_08080	AHA_1451	ASA_2914	-	B565_2734
		flpB	-	AHML_08085	AHA_1452	ASA_2913*	-	B565_2733
		flpC	-	AHML_08090	AHA_1453	ASA_2912	-	B565_2732
		flpD	-	AHML_08095	AHA_1454	ASA_2911	-	B565_2731
		flpE	-	AHML_08100	AHA_1455	ASA_2910	-	B565_2730
		flpF	-	AHML_08105	AHA_1456	ASA_2909	-	B565_2729
		flpG	-	AHML_08110	AHA_1457	ASA_2908*	-	B565_2728
		flpH	-	AHML_08115	AHA_1458	ASA_2907	-	B565_2727

		flpI	-	AHML_08120	AHA_1459	ASA_2906*	-	B565_2726
		flpJ	-	AHML_08125	AHA_1460	ASA_2905	-	B565_2725
		flpK	-	AHML_08130	AHA_1461	ASA_2904	-	B565_2724
		flpL	-	AHML_08135	AHA_1462	ASA_2903	-	B565_2723
	Lateral flagella	flgC	-	-	-	ASA_0364	-	-
		flgE	-	-	-	ASA_0366	-	-
		flgI	-	-	-	ASA_0370	-	-
		flgJ	-	-	-	ASA_0371	-	-
		fliF	-	-	-	ASA_0355	-	-
		fliG	-	-	-	ASA_0356	-	-
		fliP	-	-	-	ASA_0348	-	-
		lafB	-	-	-	ASA_0379	-	-
		lafC	-	-	-	ASA_0380	-	-
		lafE	-	-	-	ASA_0382	-	-
		lafF	-	-	-	ASA_0383	-	-
		lafK	-	-	-	ASA_0353	-	-

		lafS	-	-	-	ASA_0384	-	-
		lafT	-	-	-	ASA_0385	-	-
		lafU	-	-	-	ASA_0386	-	-
		lafX	-	-	-	ASA_0381	-	-
		lfgA	-	-	-	ASA_0362	-	-
		lfgB	-	-	-	ASA_0363	-	-
		lfgF	-	-	-	ASA_0367	-	-
		lfgG	-	-	-	ASA_0368	-	-
		lfgH	-	-	-	ASA_0369	-	-
		lfgK	-	-	-	ASA_0372	-	-
		lfgL	-	-	-	ASA_0373	-	-
		lfgM	-	-	-	ASA_0361	-	-
		lfgN	-	-	-	ASA_0360	-	-
		lfhA	-	-	-	ASA_0352	-	-
		lfhB	-	-	-	ASA_0351	-	-
		lfiE	-	-	-	ASA_0354	-	-

		lfiH	-	-	-	ASA_0357	-	-
		lfiI	-	-	-	ASA_0358	-	-
		lfiJ	-	-	-	ASA_0359	-	-
		lfiM	-	-	-	ASA_0346	-	-
		lfiN	-	-	-	ASA_0347	-	-
		lfiQ	-	-	-	ASA_0349	-	-
		lfiR	-	-	-	ASA_0350	-	-
		maf-5	-	-	-	ASA_0374	-	-
	Mannose-sensitive hemagglutinin (Msh) pilus	mshA	R2X36_19750	AHML_01955	-	-	-	B565_3659
		mshB	R2X36_19755	AHML_01950	AHA_0395	-	-	B565_3660
		mshC	R2X36_19745	AHML_01960	AHA_0396	-	-	B565_3658
		mshD	R2X36_19740	AHML_01965	AHA_0397	ASA_3941	-	B565_3657
		mshE	R2X36_19770	AHML_01935	AHA_0392	-	-	B565_3663
		mshF	R2X36_19760	AHML_01945	AHA_0394	-	-	B565_3661
		mshG	R2X36_19765	AHML_01940	AHA_0393	-	-	B565_3662
		mshI1	R2X36_19800	AHML_01905	AHA_0387	ASA_3946	-	B565_3669

		mshI	R2X36_19805	AHML_01900	AHA_0385	ASA_3947	-	B565_3670
		mshJ	R2X36_19795	AHML_01910	AHA_0388	ASA_3945	-	B565_3668
		mshK	R2X36_19790	AHML_01915	-	ASA_3944	-	B565_3667
		mshL	R2X36_19785	AHML_01920	AHA_0389	ASA_3943	-	B565_3666
		mshM	R2X36_19780	AHML_01925	AHA_0390	ASA_3942	-	B565_3665
		mshN	R2X36_19775	AHML_01930	AHA_0391	-	-	B565_3664
		mshO	R2X36_19735	AHML_01970	AHA_0398	ASA_3940	-	B565_3656
		mshP	R2X36_19730	AHML_01975	-	ASA_3939	-	B565_3655
		mshQ	R2X36_19725*	AHML_01980	AHA_0399	ASA_3938	-	B565_3654
	Polar flagella	Undetermined	R2X36_06620	AHML_07520	AHA_1389	ASA_1361	-	B565_1123
		Undetermined	-	-	AHA_4177	ASA_0151	-	-
		cheA-2	R2X36_06600	AHML_07500	AHA_1385	ASA_1357	-	B565_1119
		cheB-2	R2X36_06605	AHML_07505	AHA_1386	ASA_1358	-	B565_1120
		cheR-3	R2X36_07720	AHML_15310	AHA_2843	ASA_1488	-	B565_2607
		cheV	R2X36_07715	AHML_15315	AHA_2844	ASA_1487	-	B565_2608
		cheW	R2X36_06625	AHML_07525	AHA_1390	ASA_1362	-	B565_1124

		cheY	R2X36_06590	AHML_07490	AHA_1383	ASA_1355	-	B565_1117
		cheZ	R2X36_06595	AHML_07495	AHA_1384	ASA_1356	-	B565_1118
		flaA	R2X36_13385	AHML_09350	AHA_1698	ASA_2662	-	B565_1452
		flaB	R2X36_13380	AHML_09365	AHA_1699	ASA_2661	-	B565_1453;<br>B565_1454
		flaG	R2X36_13375	AHML_09370	AHA_1700	ASA_2660	-	-
		flaH	R2X36_13370	AHML_09375	AHA_1701	ASA_2659	-	B565_1455
		flaJ	R2X36_13365	AHML_09380	AHA_1702	ASA_2658	-	B565_1456
		flgA	R2X36_07710	AHML_15320	AHA_2845	ASA_1486	-	B565_2609
		flgB	R2X36_07725	AHML_15305	AHA_2842	ASA_1489	-	B565_2606
		flgC	R2X36_07730	AHML_15300	AHA_2841	ASA_1490	-	B565_2605
		flgD	R2X36_07735	AHML_15295	AHA_2840	ASA_1491	-	B565_2604
		flgE	R2X36_07740	AHML_15290	AHA_2839	ASA_1492	-	B565_2603
		flgF	R2X36_07745	AHML_15285	AHA_2838	ASA_1493	-	B565_2602
		flgG	R2X36_07750	AHML_15280	AHA_2837	ASA_1494	-	B565_2601
		flgH	R2X36_07755	AHML_15275	AHA_2836	ASA_1495	-	B565_2600

		flgI	R2X36_07760	AHML_15270	AHA_2835	ASA_1496	-	B565_2599
		flgJ	R2X36_07765	AHML_15265	AHA_2834	ASA_1497	-	B565_2598
		flgK	R2X36_07770	AHML_15260	AHA_2833	ASA_1498	-	B565_2597
		flgL	R2X36_07775	AHML_15255	AHA_2832	ASA_1499*	-	B565_2596
		flgM	R2X36_07705	AHML_15325	AHA_2846	ASA_1485	-	B565_2610
		flgN	R2X36_07700	AHML_15330	AHA_2847	ASA_1484	-	B565_2611
		flhA	R2X36_06570	AHML_07470	AHA_1379	ASA_1351	-	B565_1113
		flhB	R2X36_06565	AHML_07465	AHA_1378	ASA_1350	-	B565_1112
		flhF	R2X36_06575	AHML_07475	AHA_1380	ASA_1352	-	B565_1114
		flhG	R2X36_06580	AHML_07480	AHA_1381	ASA_1353	-	B565_1115
		fliA	R2X36_06585	AHML_07485	AHA_1382	ASA_1354	-	B565_1116
		fliE	R2X36_06495	AHML_07395	AHA_1364	ASA_1336	-	B565_1098
		fliF	R2X36_06500	AHML_07400	AHA_1365	ASA_1337	-	B565_1099
		fliG	R2X36_06505	AHML_07405	AHA_1366	ASA_1338	-	B565_1100
		fliH	R2X36_06510	AHML_07410	AHA_1367	ASA_1339	-	B565_1101
		fliI	R2X36_06515	AHML_07415	AHA_1368	ASA_1340	-	B565_1102

		fliJ	R2X36_06520	AHML_07420	AHA_1369	ASA_1341	-	B565_1103
		fliK	R2X36_06525	AHML_07425	AHA_1370	ASA_1342	-	B565_1104
		fliL	R2X36_06530	AHML_07430	AHA_1371	ASA_1343	-	B565_1105
		fliM	R2X36_06535	AHML_07435	AHA_1372	ASA_1344	-	B565_1106
		fliN	R2X36_06540	AHML_07440	AHA_1373	ASA_1345	-	B565_1107
		fliO	R2X36_06545	AHML_07445	AHA_1374	ASA_1346	-	B565_1108
		fliP	R2X36_06550	AHML_07450	AHA_1375	ASA_1347	-	B565_1109
		fliQ	R2X36_06555	AHML_07455	AHA_1376	ASA_1348	-	B565_1110
		fliR	R2X36_06560	AHML_07460	AHA_1377	ASA_1349	-	B565_1111
		flmD	-	AHML_09415	AHA_4179	ASA_0149	-	-
		flmH	-	AHML_11680	AHA_4175	ASA_0153	-	-
		flrA	R2X36_07805	AHML_15225	AHA_2826	ASA_1505*	-	B565_2590
		flrB	R2X36_07810	AHML_15220	AHA_2825	ASA_1506	-	B565_2589
		flrC	R2X36_07815	AHML_15215	AHA_2824	ASA_1507	-	B565_2588
		maf-1	R2X36_13355	-	AHA_1703	-	-	B565_1458
		maf-2	-	-	AHA_4181	ASA_0147	-	-

		motX	R2X36_03000	AHML_03390	AHA_0660	ASA_0660	-	B565_3496
		motY	R2X36_08910	AHML_14015	AHA_2642	ASA_2493	-	-
		nueA	-	AHML_14845	AHA_4178	ASA_0150	-	-
		nueB	-	AHML_09410	AHA_4180	ASA_0148	-	-
		pomA2	R2X36_05420	AHML_17725	AHA_3318	ASA_0993	-	B565_3201
		pomA	R2X36_06610	AHML_07510	AHA_1387	ASA_1359	-	B565_1121
		pomB2	R2X36_05425; R2X36_09275	AHML_17720	AHA_3317	ASA_1737	-	B565_3200
		pomB	R2X36_06615	AHML_07515	AHA_1388	ASA_1360	-	B565_1122
	Tap type IV pili	tapA	R2X36_19010	AHML_20500	AHA_3868	ASA_0414	-	-
		tapB	R2X36_19015	AHML_20505	AHA_3869	ASA_0413	-	B565_0359
		tapC	R2X36_19020	AHML_20510	AHA_3870	ASA_0412*	-	B565_0358
		tapD	R2X36_19025	AHML_20515	AHA_3871	ASA_0411	-	B565_0357
		tapF	R2X36_09160	AHML_09695	AHA_1757	ASA_2601*	-	B565_2370
		tapM	R2X36_06010	AHML_17120	AHA_3194	ASA_1120	-	B565_0964
		tapN	R2X36_06015	AHML_17115	AHA_3193	ASA_1121	-	B565_0965
		tapO	R2X36_06020	AHML_17110	AHA_3192	ASA_1122	-	B565_0966

		tapP	R2X36_06025	AHML_17105	AHA_3191	ASA_1123	-	B565_0967
		tapQ	R2X36_06030	AHML_17100	AHA_3190	ASA_1124	-	B565_0968
		tapT	R2X36_02855	AHML_19295	AHA_3665	ASA_3632	-	B565_3513
		tapU	R2X36_02850	AHML_19300	AHA_3666	ASA_3633	-	B565_3512
		tapV	R2X36_08575	AHML_14210	AHA_2681	ASA_2532	-	B565_1560
		tapW	R2X36_08235	AHML_14530	AHA_2739	ASA_1634	-	B565_1398
		tapY1	-	AHML_03545	AHA_0690	ASA_0692	-	B565_3465
		tapY2	-	-	-	-	-	B565_3464
		tppA	-	AHML_03550	AHA_0691	ASA_0688	-	B565_3469
		tppB	-	AHML_03530	AHA_0687	ASA_0689	-	B565_3468
		tppC	-	-	-	ASA_0690	-	B565_3467
		tppD	-	-	-	ASA_0691	-	B565_3466
		tppE	-	AHML_03525	AHA_0686	ASA_0693	-	B565_3462
		tppF	R2X36_02665	AHML_19440	AHA_3693	ASA_3659	-	B565_3457
	Type I fimbriae	fimA	-	AHML_02665	AHA_0519	ASA_3725	-	B565_0475
		fimC	-	AHML_02675	AHA_0521	ASA_3727	-	B565_0477

		fimD	-	AHML_02680	AHA_0522	ASA_3728	-	B565_0478
		fimE	-	AHML_02685	AHA_0523	ASA_3729	-	B565_0479
		fimF	-	AHML_02690	AHA_0524	ASA_3730	-	B565_0480
	LPS O-antigen (P. aeruginosa)(Pseudomonas)		R2X36_07480; R2X36_07510	-	-	-	-	-
Secretion system	T2SS	exeA	R2X36_18525	AHML_20105	AHA_3785	ASA_0515	-	B565_2887
		exeB	R2X36_18530	AHML_20110	AHA_3786	ASA_0514	-	B565_2888
		exeC	R2X36_18465	AHML_02905	AHA_0568	ASA_3774	-	B565_2889
		exeD	R2X36_18460	AHML_02910	AHA_0569	ASA_3775	-	B565_2890
		exeE	R2X36_18455	AHML_02915	AHA_0570	ASA_3776	-	B565_2891
		exeF	R2X36_18450	AHML_02920	AHA_0571	ASA_3777	-	B565_2892
		exeG	R2X36_18445	AHML_02925	AHA_0572	ASA_3778	-	B565_2893
		exeH	R2X36_18440	AHML_02935	AHA_0573	ASA_3779	-	B565_2894
		exeI	R2X36_18435	AHML_02940	AHA_0574	ASA_3780	-	B565_2895
		exeJ	R2X36_18430	AHML_02945	AHA_0575	ASA_3781	-	B565_2896

		exeK	R2X36_18415	AHML_02950	AHA_0576	ASA_3782	-	B565_2897
		exeL	R2X36_18410	AHML_02955	AHA_0577	ASA_3783	-	B565_2898
		exeM	R2X36_18405	AHML_02960	AHA_0578	ASA_3784	-	B565_2899
		exeN	R2X36_18400	AHML_02965	AHA_0579	ASA_3785	-	B565_2900
		tapD	R2X36_19025*	AHML_20515	AHA_3871	ASA_0411	-	B565_0357
	T3SS	acr1	-	-	-	-	ASA_P5G074	-
		acr2	-	-	-	-	ASA_P5G073	-
		acrG	-	-	-	-	ASA_P5G068	-
		acrH	-	-	-	-	ASA_P5G066	-
		acrR	-	-	-	-	ASA_P5G069	-
		acrV	-	-	-	-	ASA_P5G067	-
		aexT	-	-	-	ASA_4266	-	-
		aopB	-	-	-	-	ASA_P5G065	-
		aopD	-	-	-	-	ASA_P5G064	-
		aopH	-	-	-	-	ASA_P5G009	-
		aopN	-	-	-	-	ASA_P5G075	-

		aopO	-	-	-	-	ASA_P5G098	-
		aopX	-	-	-	-	ASA_P5G084*	-
		ascB	-	-	-	-	ASA_P5G058	-
		ascC	-	-	-	-	ASA_P5G057	-
		ascD	-	-	-	-	ASA_P5G056	-
		ascE	-	-	-	-	ASA_P5G055	-
		ascF	-	-	-	-	ASA_P5G054	-
		ascG	-	-	-	-	ASA_P5G053	-
		ascH	-	-	-	-	ASA_P5G052	-
		ascI	-	-	-	-	ASA_P5G051	-
		ascJ	-	-	-	-	ASA_P5G050	-
		ascK	-	-	-	-	ASA_P5G049	-
		ascL	-	-	-	-	ASA_P5G048	-
		ascN	-	-	-	-	ASA_P5G076	-
		ascO	-	-	-	-	ASA_P5G077	-
		ascP	-	-	-	-	ASA_P5G078	-

		ascQ	-	-	-	-	ASA_P5G079	-
		ascR	-	-	-	-	ASA_P5G080	-
		ascS	-	-	-	-	ASA_P5G081	-
		ascT	-	-	-	-	ASA_P5G082	-
		ascU	-	-	-	-	ASA_P5G083	-
		ascV	-	-	-	-	ASA_P5G070	-
		ascX	-	-	-	-	ASA_P5G072	-
		ascY	-	-	-	-	ASA_P5G071	-
		ati1	-	-	-	-	ASA_P5G046	-
		ati2	-	-	-	-	ASA_P5G045	-
		exsA	-	-	-	-	ASA_P5G060	-
		exsB	-	-	-	-	ASA_P5G061	-
		exsC	-	-	-	-	ASA_P5G063	-
		exsD	-	-	-	-	ASA_P5G059	-
		exsE	-	-	-	-	ASA_P5G062	-
		sycH	-	-	-	-	ASA_P5G008	-

		sycO	-	-	-	-	ASA_P5G097	-
		sycX	-	-	-	-	ASA_P5G085	-
	T6SS	Undetermined	R2X36_12695	-	AHA_1847	ASA_2456	-	-
		atsA	R2X36_12790	-	AHA_1828	-	-	-
		atsB	R2X36_12785	-	AHA_1829	-	-	-
		atsC	R2X36_12780	-	AHA_1830	-	-	-
		atsD	R2X36_12775	-	AHA_1831	ASA_0454	-	-
		atsG	R2X36_12760	-	AHA_1834	ASA_2468	-	-
		atsH	R2X36_12755	-	AHA_1835	ASA_2467	-	-
		atsI	R2X36_12750	-	AHA_1836	ASA_2466	-	-
		atsJ	R2X36_12745	-	AHA_1837	ASA_2465	-	-
		atsK	R2X36_12740	-	AHA_1838	ASA_2464	-	-
		atsL	R2X36_12735	-	AHA_1839	ASA_2463	-	-
		atsP	R2X36_12715	-	AHA_1843	ASA_2459	-	-
		atsQ	R2X36_12710	-	AHA_1844	-	-	-
		atsS	R2X36_12700	-	AHA_1846	ASA_2457	-	-

		clpV1	R2X36_12725	-	AHA_1841	ASA_2461	-	-	
		dotU	R2X36_12730	-	AHA_1840	ASA_2462	-	-	
		hcp1	R2X36_12805	AHML_05970	AHA_1118	-	-	-	
		hcp	R2X36_16020	AHML_10025	AHA_1826	-	-	-	
		vasH	R2X36_12720	-	AHA_1842	ASA_2460	-	-	
		vasK/atsR	R2X36_12705	-	AHA_1845	-	-	-	
		vgrG1	R2X36_16010	AHML_05975	AHA_1119	-	-	-	
		vgrG2	R2X36_12795	AHML_10030	AHA_1827	-	-	-	
		vgrG3	R2X36_12690	-	AHA_1848	-	-	-	
		vipA	R2X36_12770	-	AHA_1832	ASA_2470	-	-	
		vipB	R2X36_12765	-	AHA_1833	-	-	-	
Toxin	Aerolysin AerA/Cytotoxic enterotoxin Act	aerA/act	-	AHML_02265	AHA_0438	ASA_3906	-	B565_3626	
	Extracellular hemolysin AHH1	ahh1	-	AHML_08400	AHA_1512	ASA_2854	-	-	

	Heat-stable cytotonic enterotoxin	ast	-	AHML_04100	AHA_0804	-	-	-
	Hemolysin HlyA	hlyA	R2X36_07895	AHML_15145	AHA_2809	ASA_1523	-	B565_2574
	Hemolysin III	Undetermined	R2X36_04325	AHML_18530	AHA_3493	ASA_0824	-	B565_0799
	The repeat in toxin (RTX)	rtxA	-	AHML_07370	AHA_1359	-	-	-
		rtxB	-	-	AHA_1356	-	-	-
		rtxC	-	-	AHA_1358	-	-	-
		rtxD	-	-	AHA_1355	-	-	-
		rtxE	-	-	AHA_1354	-	-	-
		rtxH	-	-	AHA_1357	-	-	-
	Thermostable hemolysin (TH)	Undetermined	R2X36_05890	AHML_17235	AHA_3217	ASA_1096	-	B565_0938
Antiphagocytosis	Capsular polysaccharide(Vibrio)	wbjD/wecB	R2X36_13505	-	-	-	-	-
		wecA	R2X36_07560	-	-	-	-	-
		wecC	R2X36_13510	-	-	-	-	-
Fimbrial adherence	Bcf(Salmonella)	bcfA	R2X36_08640	-	-	-	-	-
		bcfB	R2X36_08645	-	-	-	-	-

determinants								
Glycosylation system	O-linked flagellar glycosylation(Campylobacter)	neuB2	R2X36_07485	-	-	-	-	-
Immune evasion	Capsule(Acinetobacter)		R2X36_07470; R2X36_07475; R2X36_07495; R2X36_07535; R2X36_07540; R2X36_07545; R2X36_16675	-	-	-	-	-
	Capsule(Streptococcus)	rmlA	R2X36_07570	-	-	-	-	-
Serum resistance	LPS rfb locus(Klebsiella)	rmlD	R2X36_07565	-	-	-	-	-
Stress adaptation	Catalase-peroxidase(Mycobacterium)	katG	R2X36_09500	-	-	-	-	-

Notes: -, negative

* Identified by Blastn

Table saved from VFDB (http://www.mgc.ac.cn/VFs/) [Fri Nov 10 17:26:28 2023]
